# On the Enhancement of the Long-Term Washability of e-Textile Realized with Electrically Conductive Graphene-Based Inks

**DOI:** 10.3390/polym17070904

**Published:** 2025-03-27

**Authors:** Ilaria Improta, Gennaro Rollo, Giovanna Giuliana Buonocore, Simona Del Ferraro, Vincenzo Molinaro, Gianni D’Addio, Anna De Rosa, Marino Lavorgna

**Affiliations:** 1 Institute of Polymers, Composites and Biomaterials, National Research Council, 80055 Portici, Italy; ilariaimprota@cnr.it (I.I.); giovannagiuliana.buonocore@cnr.it (G.G.B.); marino.lavorgna@cnr.it (M.L.); 2 INAIL—DiMEILA—Laboratory of Ergonomics and Physiology, 00078 Monte Porzio Catone, Italy; s.delferraro@inail.it (S.D.F.); v.molinaro@inail.it (V.M.); 3 Bioengineering Unit, Institute of Care and Scientific Research Maugeri, 82037 Telese, Italy; gianni.daddio@icsmaugeri.it (G.D.); or anna.derosa004@studenti.uniparthenope.it (A.D.R.); 4 Engineering Department, University of Naples Parthenope, 80133 Napoli, Italy

**Keywords:** coating, thermoplastic polyurethane, conductive textile, e-textile, electrical conductivity, graphene

## Abstract

This research explores the development of highly durable flexible electronic textiles (e-textiles) for wearable electronics, focusing on improving their washability and performance. A conductive graphene-based ink was screen-printed onto a polyester textile. Water-based polyurethane (PU) coatings with variable crosslinker ratios and thickener were applied to solve washability issues. The results show that the PU coatings significantly enhanced the electrical stability and durability of the printed pathways after multiple washing cycles. The conductivity remained intact after 120 washing cycles, indicating that the final properties of the e-textile, which contained 6 wt% thickener and 3 wt% crosslinker, provided effective water protection. The results highlight the promise of these coated e-textiles for wearable electronics applications, especially in the occupational and healthcare sectors, where long-term flexibility and washability are critical.

## 1. Introduction

The growing interest in wearable technology has spurred substantial innovation in smart fabrics and advanced materials, especially for monitoring vital human metrics. [1,2,3]. Recent advances in printable inks based on 2D materials have created new opportunities to integrate electrical sensors directly into textiles, enhancing the usability and comfort of wearable technology [4,5,6]. In this context, graphene has attracted considerable attention due to its remarkable properties, such as excellent conductivity (3000–5000 W/m·K), high Young’s modulus (≈1 TPa), optical transparency (≈97.7%), and high carrier mobility (≈10,000 cm^2^/V·s) [7,8]. Graphene electrodes printed on textiles offer effective and adaptable options for continuous health monitoring [8,9,10].

The application of fabric-printed graphene, i.e., in electrocardiogram (ECG) monitoring, is considered a major step towards wearable medical technology that is less intrusive and more comfortable [11,12,13].

However, despite the promising advantages of graphene-based printed electronics, several challenges remain to ensure the durability and stability of conductive pathways on textiles [14,15,16].

Typically, graphene-based inks are water-based and environmentally friendly. Wash resistance is a critical factor for the practical integration of conductive inks in wearable e-textiles. In fact, this issue limits their practical applicability in wearable devices, where performance must be preserved even after multiple wash cycles. The use of protective coatings becomes essential to maintain conductivity and long-term stability [17].

The washing and wear resistance of graphene inks applied to fabrics are the two main disadvantages. In fact, these inks are susceptible to degradation from mechanical stress during washing cycles and exposure to moisture. The printed conductive paths lose electrical performance if not properly protected [18,19,20,21]. To address these challenges, advanced coating strategies are necessary to improve the durability and reliability of printed graphene circuits on textiles [22]. Polymer coatings can be used to maintain the elasticity and permeability of the fabric while improving adhesion, water resistance, and mechanical strength. In this context, polyurethane (PU) is one of the most promising materials with these characteristics. However, conventional PU coatings tend to absorb significantly into the textile fibers, resulting in uneven protection of the conductive pathways [23,24,25].

To mitigate this issue, the present research explores the formulation of waterborne coatings incorporating polyurethane-based thickeners and aliphatic isocyanurate crosslinkers. These additives modify the viscosity and crosslinking density of the PU matrix, creating a uniform protective layer that enhances the tailoring of the coating and increases washability and conductivity retention. In particular, the urethane-based thickener increases the density of the PU network, while the isocyanurate crosslinker forms inter-chain links that reduce polymer mobility and improve water resistance [26,27]. The combined effect of these components, in specific proportions, represents a novel approach to stabilizing graphene-based printed electronics on polyester fabric textiles by screen-printing.

To understand the effect of the coating formulation on the performance of the printed conductors on textiles, the morphology, wetting, electrical and washing properties of graphene conductors, and the effect of the pathway width were correlated with physiological monitoring. This research highlights the importance of the coating formulation and investigates the effectiveness of waterborne green polyurethane coatings. The combined properties of the two additives in different proportions, in the ratio identified, represent an element of novelty within the state of the art. The ability to effectively coat and protect conductive pathways printed on textiles with water-based inks from washing enables wearable e-textiles.

## 2. Materials and Methods

A screen-printing machine (K-SER1 EVOs by KEY, Pescara, Italy) was used to apply the ink (GUP^®^ C-Paste kindly provided by GrapheneUP, Studeněves, Czech Republic) onto 100% shuttle-woven polyester previously treated for dyeing.

Conductive pathways with a length of 180 mm and variable widths of 5, 3, and 1 mm were used. After each deposition, the textiles were cured at 120 °C for 3 min. The screen-printing and ink curing process was repeated 3 times, obtaining 3 layers of ink (Figure S1). This was carried out to maximize the electrical conductivity, as reported in the literature [4]. The ink was deposited by screen-printing following the direction of the warp.

After the ink was cured, the polyurethane coating was applied to cover the ink pathways (top and bottom of the fabric in order to prevent water contact with the water-soluble ink). The average thickness of the coating was approximately 15 µm.

Coatings were obtained by mixing water-based POLYTECH BIO E02 thermoplastic polyurethane (TPU) with different amounts of CROSSTECH 655 aliphatic polyisocyanate crosslinker and VIXTECH 872 water-based urethane thickener (kindly provided by ICAP-SIRA Chemicals and Polymers, Milano, Italy). These two additives are commercially indicated to improve TPU processing conditions. The technical data sheets for each additive indicate that CROSSTECH 655 can be used at levels between 1 and 6 wt%, while VIXTECH 872 can be used at levels between 0.5 and 3 wt%. To find the satisfactory conditions for the screen-printing deposition, starting from the minimum percentages indicated in the technical data sheets of the materials, a step-by-step evaluation of the minimum percentage concentration was required. For this reason, the first coating formulation, T1 (Table 1), was a composition of 2 wt% of crosslinker and 2.5 wt% of thickener; sample T2 contained an average amount (2.5 wt% of crosslinker and 2.5 wt% of thickener, in order to consider a ratio of 1:1); and sample T3 included the maximum amount for each material (corresponding to a percentage of 6 wt% of crosslinker and 3 wt% of thickener). To identify the different prepared systems, the following nomenclature was used: PE/Ink/T*x*/*y*, where *x* is 1, 2, and 3 according to Table 1, and *y* is 5, 3, and 1, expressed in mm, representing the width of the deposited pathways.

### 2.1. Fourier-Transform Infrared Spectroscopy (FT-IR) Analysis

Using a Frontier FT-IR/NIR spectrometer (PerkinElmer, Hopkinton, MA, USA) in the Attenuated Total Reflectance (ATR) mode, the FT-IR spectra of the TPU-based films at room temperature were recorded in the range from 650 to 4000 cm^−1^. The results were the average of 64 scans with spectra acquired at a 4 cm^−1^ resolution. TPU films with different percentages of crosslinker and thickener were tested. The Origin-Pro 8.5.0 SR1 software was used to evaluate the spectra.

### 2.2. Wettability Properties

The angle that forms at the point where a liquid and a flat surface meet is known as the contact angle. Surfaces that have a water contact angle of more than 90° are hydrophobic, whereas surfaces that have an angle of less than 90° are hydrophilic [28].

The measurements of the static contact angle were carried out using an Optical Contact Angle instrument (OCA 20 by Dataphysics, Filderstadt, Germany), dispensing a 1 µL drop to assess the wettability of the surface.

Water (polar solvent) and diiodiomethane (apolar solvent) were dropped onto at least 10 different sites of the tested samples, and the static contact angle was reported as the average value of each measurement. A roll-coating assembly with a roll bar of 50 µm was used to obtain thin-layer films for each composition.

### 2.3. Scanning Electron Microscopy Analysis (SEM)

The morphology was investigated using scanning electron microscopy (SEM) (Quanta 200 FEG, FEI, Hillsboro, OR, USA) on samples previously coated with a ~10 nm layer of an Au-Pd alloy (Emitech K575, Quorum Technologies Ltd., Lewes, UK) and mounted on aluminum stubs using carbon adhesive discs.

### 2.4. Atomic Force Microscopy Analysis (AFM)

The surface morphology of the films was analyzed using atomic force microscopy (AFM Nanosurf with C3000 Controller, LOT Quantum Design s.r.l., Rome, Italy). The soft tapping mode stabilized by amplitude-modulation feedback was used to acquire topographies. The obtained images were evaluated by the Gwyddion software v2.66.

In the analysis of the surface roughness, the parameters R_a_ (average roughness), R_z_ (average maximum height of the profile), and S_m_ (mean spacing of profile irregularities) were calculated for three samples using 10 topography images (20 × 20 μm^2^). R_a_ represents the absolute mean deviation of the surface from the mean profile but does not effectively differentiate surfaces with significant variations in peak and valley distribution or height. Therefore, R_z_, which focuses on the height difference between peaks and valleys, was also considered, as it provides more information about large variations. S_m_ measures the average distance between peaks, helping to compare surfaces with frequent small peaks versus larger, more spaced ones. This allows for assessing not only peak and valley heights but also the frequency and distribution of the surface roughness.

### 2.5. Tensile Tests

The mechanical properties of the graphene-coated fabrics with different coatings were evaluated using a dynamic mechanical analysis instrument (DMA Q800, TA Instruments, New Castle, DE, USA) in a uniaxial test setup with a preload of 0.1 N at a fixed temperature of 25 C. The loading rate was set at 0.5 N/min, and the specimen was strained to 18 N, and the values reported are the average of ten replicates [21]. Measurements were taken in the direction of the warp.

### 2.6. Electrical Resistance, Resistivity, and Washability Tests

The resistivity of the printed ink was assessed using a two-probe tester (Multimeter 34401A 6½, Agilent Technologies, Santa Clara, CA, USA), varying the deposition width of the conductive pathways. The resistivity was then calculated according to Ohm’s second law:

(1) $$\rho =R\cdot \frac{S}{l}$$

where ρ is the resistivity (Ω·mm), *R* is the electrical resistance (Ω), *S* is the cross-section (mm^2^), and *l* is the length (mm).

The electrical resistance tests were carried out at a distance of 10 mm, and the values reported were averaged over the three conductive pathways prepared. Samples were replicated three times. The reported values represent the average of the measurements from all replicates.

Resistivity and electrical resistance tests with the coating were carried out using a stainless-steel conductive ribbon (5 mm wide) fixed to the ink with a conductive paste. The conductive tape was attached to the end of the printed pathways so that the resistivity value could be read even after applying the insulating coating, as shown in Figure S2. To evaluate the washability of textile-based conductive pathways, a standard washing test was established by ISO 6330 [29]. The conductive pathways were tested for 120 cycles of soap washing with 5 g/L soap stirred at 500 rpm for an hour at 30 °C and dried at room temperature.

### 2.7. Piezoresistive Characterization

Piezoresistive behavior consists of the variation in electrical resistance when a tensile or compressive force is varied. This allows for electrical resistance values to be correlated with corresponding strain or load values [30,31]. The samples were tested under strain control using a universal testing machine (CMT 4304, Sans Materials Testing Co. Ltd., Shenzhen, China). Bending/unbending cycles were performed with a 15 mm deformation at 10 mm/min. The electrical signal was monitored and recorded with a multimeter (Keysight 34401A 6½ Digit Multimeter, Agilent Technologies, Santa Clara, CA, USA, 2006) controlled by a homemade LabVIEW program. The results were evaluated to calculate the Gauge Factor (GF), which is the transduction factor defined as the ratio of the fractional change in electrical resistance (ΔR = R − R_0_, where R is the electrical resistance value at maximum deformation and R_0_ is the electrical resistance without deformation) to the fractional change in length (strain, ε), expressed according to the following formula [32,33]:

(2) $$GF=\frac{∆R/R_{0}}{\varepsilon }$$

### 2.8. Measurement Chain for ECG Signal Acquisition

A MedSim 300B (Fluke Corporation, Everett, WA, USA) patient simulator was used to acquire the echocardiographic ECG signal. The ECG signal for this study was obtained using an Arduino Uno and a Gravity Heart Rate Monitor sensor. The ECG signal was obtained using BITalino (r)evolution (Plux wireless biosignals, Lisbon, Portugal) connected to the patient simulator [34]. The BITalino data were acquired using a PC running the Open Signal software v2.2.5 (Plux wireless biosignals, Lisbon, Portugal), and the signals from the Arduino UNO board were acquired using a PC running the CoolTerm software v. 2.1.1.1359.

## 3. Results and Discussion

### 3.1. FT-IR Analysis

**Figure 1:**
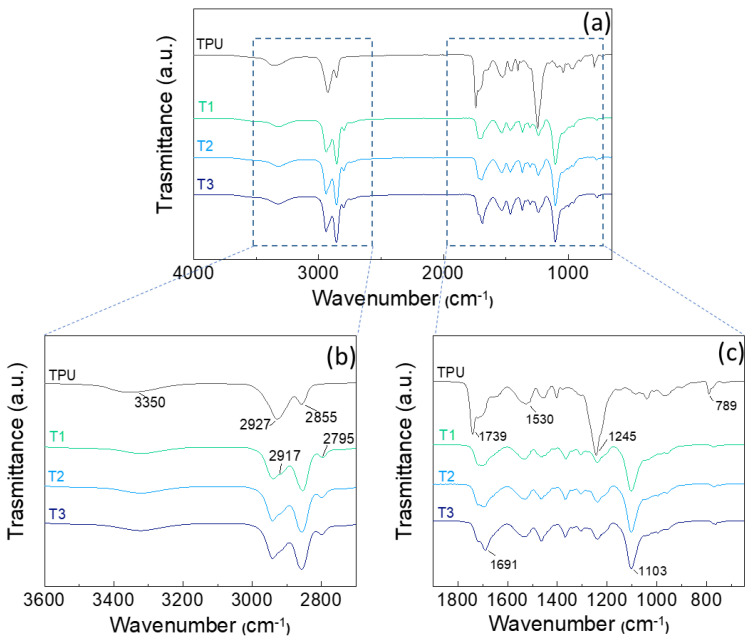
FT-IR spectra of (**a**) TPU (black) and coatings T1 (green), T2 (light blue), and T3 (navy blue), ranging between (**b**) 3600–2600 cm^−1^ and (**c**) 1900–600 cm^−1^.

Figure 1 shows the comparison between the spectra of the TPU films with different crosslinker and thickener ratios. The N-H stretching mode is responsible for the absorption peak at 3320 cm^−1^. The peak at 2935 cm^−1^ is due to the stretching of asymmetric methylene (-CH_2_), while the 2856 and 2798 cm^−1^ peaks are responsible for symmetric methylene. The shoulder at 2917 cm^−1^ is due to adding the polyurethane thickener in systems T1, T2, and T3, leading to the stretching of -CH_2_ and splitting of the symmetric and asymmetric stretches (Figure 1b) [35].

The peak at 1739 cm^−1^ is attributed to the carbonyl group (C=O) stretching mode in urethane. At 1530 and 1245 cm^−1^, amide II (*st* C-N+δ N-H) and amide III (*st* C-N+C=O) are reflected, respectively. These peaks, which are characteristic of TPU, are significantly less intense or disappear when the crosslinker and thickener are added, as both the carbonyl group and the amide groups II and III interact with the functional groups of the additives. The band at 1691 cm^−1^, which appears starting from T1 and increases in T3 (Figure 1c), is attributed to the H-bonds and the interaction with the polymeric matrix of the C=O stretching modes in the isocyanurate of the crosslinker [36,37]. The 1000–1200 cm^−1^ signals are due to polyoxyethylene (POE) units of the thickener with urethane groups and terminal hydrophobic groups contributing to a higher intensity of C-O-C stretching [38]. The absorption at 780 cm^−1^ is responsible for the bending mode of the non-planar CO-O-C in the urethane structure [39,40].

### 3.2. Morphological and Topographical Analysis

The morphology of the surfaces without and with the polyurethane coatings was observed using SEM analysis, and the uncoated and coated fabrics before and after washing and drying cycles were compared.

**Figure 2:**
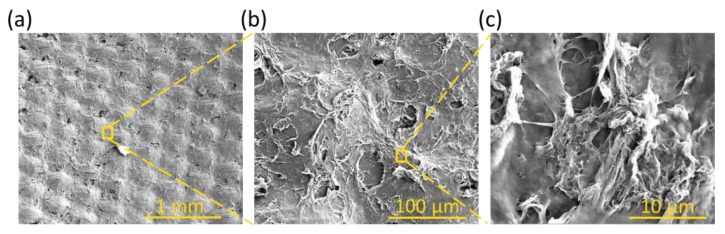
SEM images of PE/Ink of ink at magnifications of (**a**) 100×, (**b**) 800×, and (**c**) 1600×.

**Figure 3:**
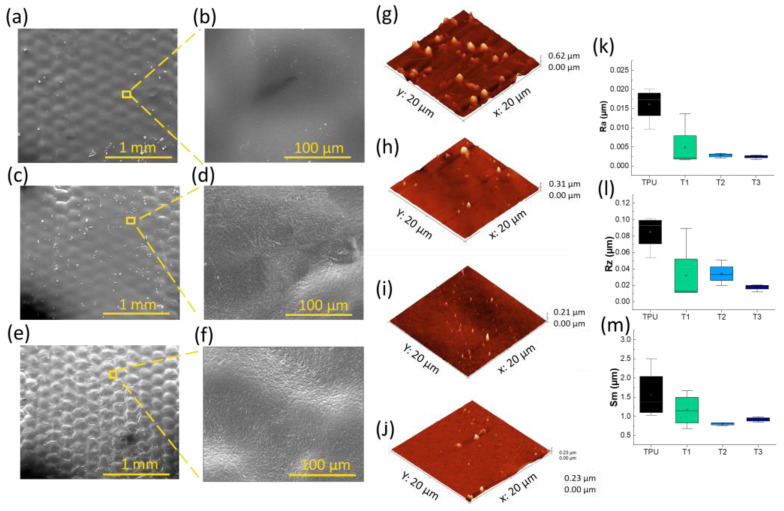
SEM images of PE fabrics (**a**,**b**) PE/Ink/T1/5, (**c**,**d**) PE/Ink/T2/5, and (**e**,**f**) PE/Ink/T3/5 at magnifications of 100× and 800×. AFM images of pristine (**g**) PU, (**h**) T1, (**i**) T2, and (**j**) T3. (**k**) Average roughness (R_a_), (**l**) average maximum height of the profile (R_z_), and (**m**) mean spacing of profile irregularities (S_m_) for pristine PU (black), T1 (green), T2 (light blue), and T3 (navy blue) films.

In particular, Figure 2 shows that the uncoated samples have an irregular surface, exposing the ink with the graphene filler inside. Instead, Figure 3 shows the coated fabric surfaces. The amount of crosslinker and thickener affects the morphology of the surfaces; in particular, the surface roughness decreases as the percentage of crosslinker increases, as reported in Figure 3b,d,f. This observation is confirmed by the topographical analysis, as shown in Figure 3g–j [41]. Figure 3k–m show the distributions of the PU, T1, T2, and T3 film surface roughness parameters measured from each topography image; it can be observed that the lowest roughness values were obtained for T3, indicating a smoother surface, as indicated by R_a_, according to the contact angle observations and Wenzel model predictions. From R_z_, the roughness is given by the presence of peaks on the surface and not by the valleys. The S_m_ value shows that by increasing the crosslinker percentage, there was a decrease in the size of the peaks and a greater distribution of them on the surface.

**Figure 4:**
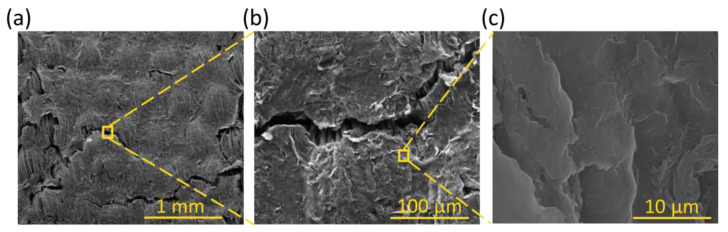
SEM images of PE/Ink after 3 h of washing at (**a**) 100×, (**b**) 800×, and (**c**) 1600× magnifications.

**Figure 5:**
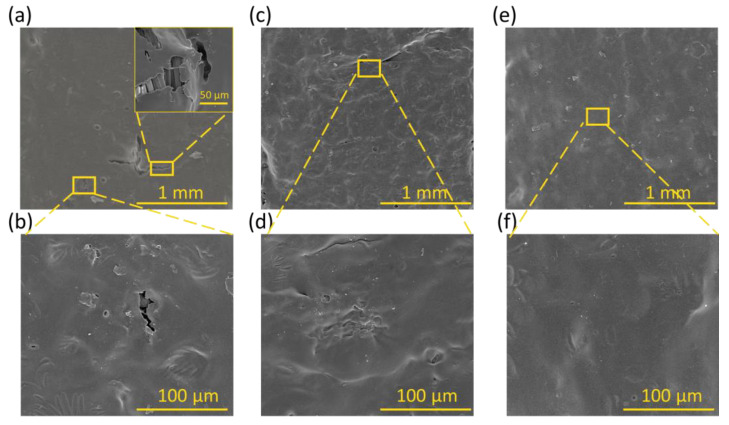
SEM images of (**a**,**b**) PE/Ink/T1/5, (**c**,**d**) PE/Ink/T2/5, and (**e**,**f**) PE/Ink/T3/5 at magnification of 100× and 800×.

Figure 4 and Figure 5 show the uncoated and coated samples subjected to washing cycles, respectively. In particular, Figure 5 shows how the uncoated ink began to peel off after 3 h of washing, leaving many parts of the PE fabric uncovered.

Figure 5 shows SEM images of the samples coated with the T1, T2, and T3 coatings. By increasing the crosslinker and thickener amounts, a more uniform surface was gradually observed. In fact, after 120 washing cycles, T1 showed an evident degradation, which also showed the substrate fibers of the fabric. On the other hand, after the same number of washing cycles, the interface between the coating and the fibers for T3 was still free of defects and without discontinuities on the surface.

### 3.3. Wettability Properties

**Figure 6:**
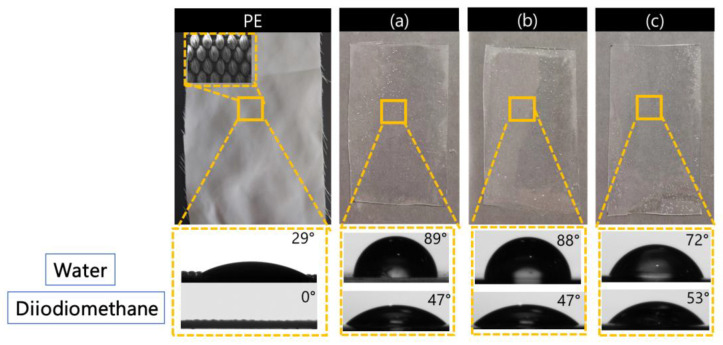
Static contact angle measurements on the polyester fabric surface and coatings (**a**) T1, (**b**) T2, and (**c**) T3 with water and diiodiomethane solvent.

Figure 6 shows the wettability of two different solvents, water and diiodomethane, evaluated by measuring the contact angle. As the percentage of crosslinker and thickener was increased, the contact angle with the aqueous solvent decreased and the contact angle with diiodiomethane increased. On the other hand, the sample without the coating showed a hydrophilic fabric surface, while the angle formed with diiodiomethane was zero.

The increased exposure of hydrophilic groups resulted in an overall wetter surface; in particular, more polyisocyanate crosslinker led to the exposure of urethane or urea polar groups, as shown in the FT-IR results in Figure 1, which interacted with the water to decrease the contact angle [42]. A smooth surface with a higher density of exposed polar groups can interact more strongly with water and can provide a larger contact area between the water droplet and the material. This effect is described by the Wenzel model, which states that water tends to spread out on a smooth surface, reducing the contact angle. Therefore, when the surface is less rough, water will adhere better, increasing wettability [43].

### 3.4. Tensile Tests

To evaluate the effect of the coating on the stretchability and flexibility of wearable smart electrical textiles, tensile experiments were conducted [44].

**Figure 7:**
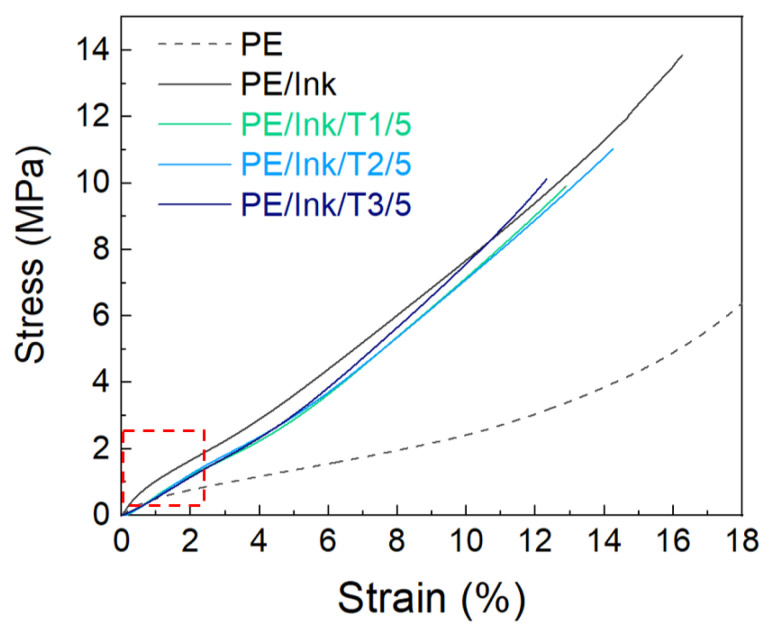
Mechanical properties of pristine PE textile (black dots) with three layers of ink (black) and coated with PU, T1 (green), T2 (light blue), and T3 (navy blue).

The initial part of the stress–strain curve, the red box in Figure 7, showed a complex variety of deformations, such as the elimination of yarn curling and straightening, followed by a rapid increase in fabric tension. A comparative analysis of the various samples was therefore carried out by calculating the tensile modulus between 6 and 8% of the stress in order to eliminate the initial phenomena characteristic of the fabric [45,46].

Table 2 shows that the pristine PE fabric had the lowest tensile modulus and the highest elongation, which increased with the application of the conductive path. The development of three-dimensional networks connecting individual fibers within the fabric increased the creep resistance of adjacent fibers, affecting their mobility and increasing the stiffness of the coated samples. The presence of additives led to the formation of new hydrogen bonds (as discussed in the FT-IR section), which affected the observed variations in mechanical properties. As can be seen from Figure 7 and the values in Table 2, the application of the coatings did not result in significant changes in the modulus compared to PE/Ink [21].

### 3.5. Electrical Analysis

**Figure 8:**
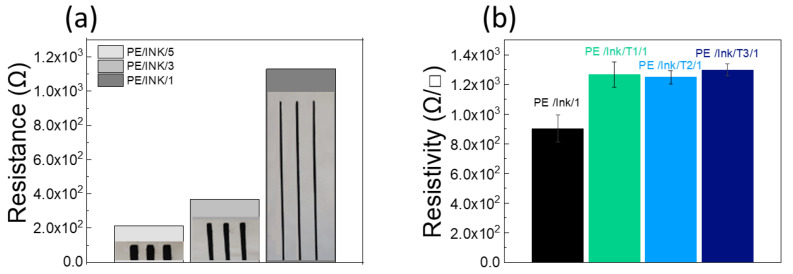
(**a**) Electrical resistance values for the different uncoated conductive line widths (5, 3, and 1 mm), and (**b**) resistivity tests on the ink fabric before and after T1, T2, and T3 deposition on 1 mm conductive line.

Figure 8a shows the electrical resistance values for the different widths of the conductive pathways. The electrical resistance of the sample with 1 mm paths differed from that of the 3 mm and 5 mm pathways by an order of magnitude and was not linear, as would be expected from Ohm’s law. This shows that in addition to the decrease in percolative paths due to the smaller conductive section for the 1 mm samples, there was also an edge effect due to the intrinsic nature of the coupling of the fabric with the ink, leading to irregular edges [47,48].

In order to assess the influence of the coating on the passage of electrical charges within the conductive pathways, resistivity tests were carried out on the fabric before and after coating. Figure 8b shows the trend for the 1 mm conductive pathways. In all cases, it was possible to measure an increase in resistivity after coating deposition (by approximately 200 Ω/◻). This behavior was due to the intercalation of the coating within the deposited ink, decreasing the connections between the FLGs.

Resistivity tests were also carried out on the samples subjected to the washing tests.

**Figure 9:**
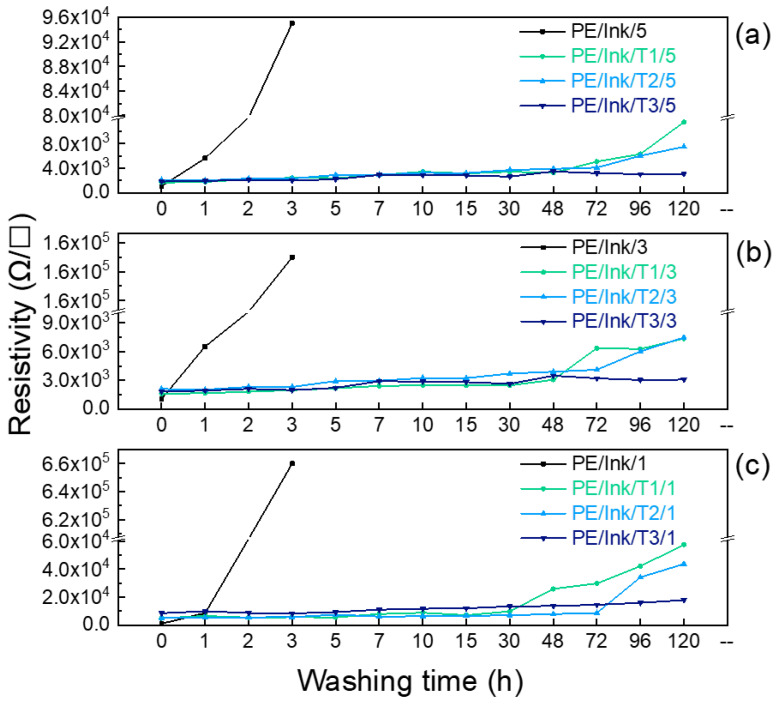
Resistivity values during 120 h washing test for electrically conductive ink line with a width of (**a**) 5 mm, (**b**) 3 mm, and (**c**) 1 mm coated with T1, T2, and T3.

**Figure 10:**
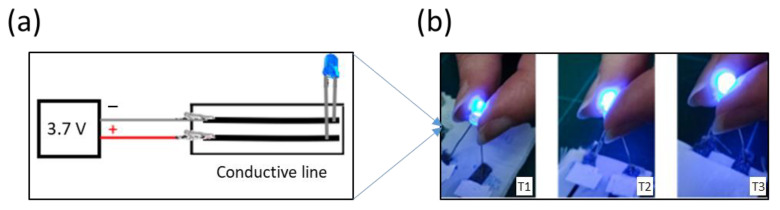
(**a**) Schematic representation of chain for checking current flow, (**b**) using a blue LED test with 1 mm conductive pathways after 120 h of washing.

The results obtained are shown in Figure 8, Figure 9 and Figure 10 for the tests on the 5, 3, and 1 mm conductive pathways coated with T1, T2, and T3.

As shown in Figure 9a, it was observed that after 3 h of washing, the uncoated ink was no longer conductive, which is consistent with the degradation phenomena observed in the SEM analysis and therefore justifies the application of a protective coating. T1 gave the highest resistivity values after 120 h. This was due to the lower crosslinker percentage, which allowed the soapy water to penetrate the coating, causing its degradation, as also observed in the SEM analysis.

Furthermore, unlike T3, T1 and T2 showed an increase in resistivity after 48 h of washing, related to the larger surface area to be coated, which increases the likelihood that the coating will deteriorate during washing and that water will be exposed to the ink and fabric.

Figure 10b shows that after 120 h of washing, the LED lit up by applying a potential difference of 3.7 V. In addition, the LED light became progressively more intense from T1 to T3, correlating with the electrical resistivity.

### 3.6. Piezoresistive and ECG Signal Acquisition Performance

Based on the results of the mechanical and washing resistance tests, performance tests were carried out in this section to assess the piezoresistive properties and the possibility of using the conductive ink coated within a measurement chain for the acquisition of an ECG signal.

**Figure 11:**
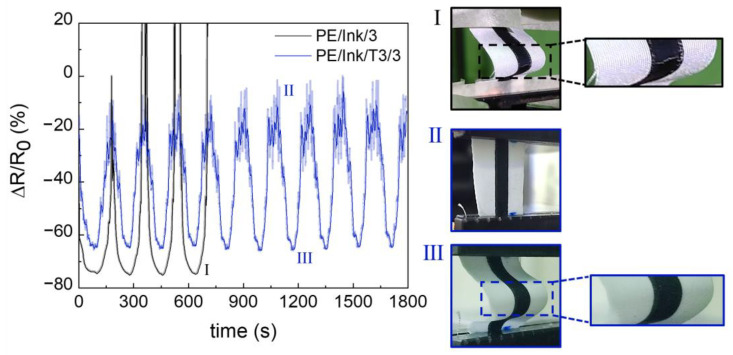
Piezoresistive behavior of PE/Ink/3 (black) and PE/Ink/T3/3 (navy blue) for bending/unbending cycles.

The piezoresistive tests showed the sensitivity of the conductive inks to bending and unbending deformation. As shown in Figure 11, the uncoated sample, PE/Ink/3, was overloaded after four cycles. The inset in Figure 11I shows a crack in the deposited ink and subsequent interruption of the conductive pathways.

On the other hand, the samples with the coating (i.e., system with the T3 coating shown in Figure 11) preserved the condition of the conductive ink and increased its durability. As shown in Figure 11III, the conductive line was free of defects after more than 10 cycles.

In addition, when the sample was completely unbended (Figure 11II), the coating particularly reduced the sensitivity and stability of the signal. The decrease in sensitivity caused by the application of the coating was quantified by the GF, using Formula (2), which was 4.94 ± 0.029 for the PE/Ink/3 system and 4.28 ± 0.054 for the PE/Ink/T3/3 system, with a percentage loss of 13%.

Therefore, the coating can be used to increase long-term usability, taking into account the high error at low strains.

**Figure 12:**
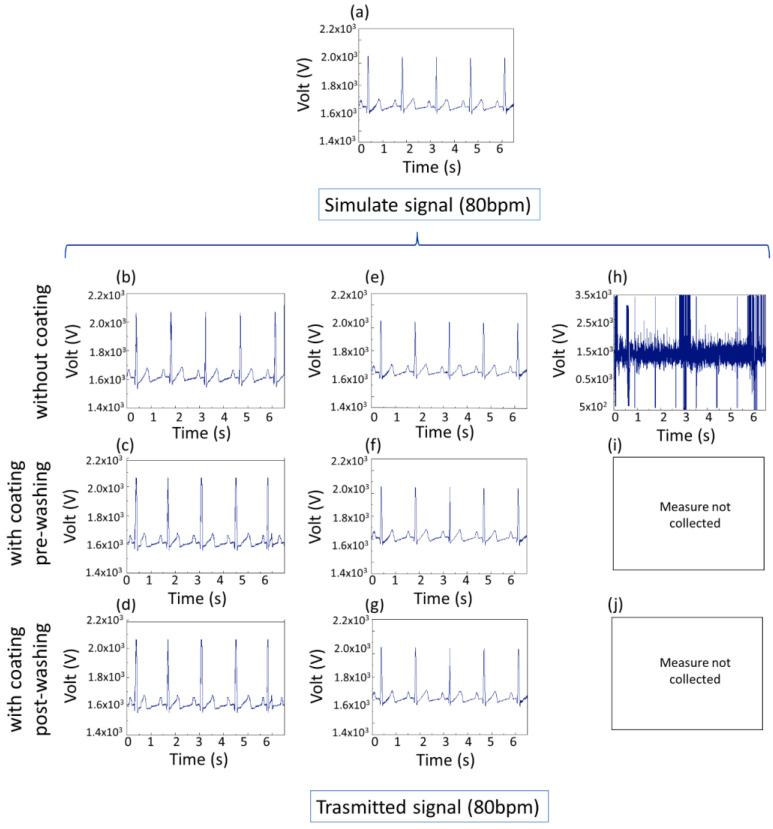
Simulated signal (**a**) and transmitted signal at 80 bpm for three different widths of conductive line coated with T3. Signals for specimens (**b**) without coating, (**c**) with coating pre-washing, and (**d**) with coating post-washing for conductive lines of 5 mm width; signals for specimens (**e**) without coating, (**f**) with coating pre-washing, and (**g**) with coating post-washing for conductive pathways of 3 mm width; signals for specimens (**h**) without coating for conductive pathways of 1 mm width. (**i**,**j**) Measure not collected.

This study found that the resistivity of the conductive pathways had a direct impact on the ECG signal quality. The acceptable threshold for effective ECG signal transmission was determined to be a resistivity below 10^4^ Ω∙mm [49]. Fabrics with a resistivity lower than this threshold provided clear ECG signals, indicating proper electrical conduction. However, when resistivity values exceeded 10⁴ Ω∙mm, the signal quality diminished significantly, becoming either excessively noisy or failing to pass through the system. Figure 12 shows the 1 mm wide conductive pathways struggling to transmit the ECG signal effectively even without a coating, indicating that narrower lines face greater challenges in maintaining conductivity for physiological monitoring applications. These findings underscore the importance of optimizing both the conductive ink formulation and the coating to ensure reliable performance in healthcare-related wearable devices.

## 4. Conclusions

This study shows how waterborne graphene-based conductive ink, appropriately coated, may be successfully used to create long-lasting, flexible, and washable e-textiles. A water-based polyurethane (PU) coating was used to overcome significant problems with the washability and long-term performance of wearable e-textiles. The role of CROSSTECH 655 aliphatic polyisocyanate crosslinker and VIXTECH 872 water-based urethane thickener was assessed at different ratios. The results suggest that the washing resistance of the coating was greatly improved by raising the concentration of thickener and crosslinker, which prevented the conductive pathways from degrading even after 120 washing cycles.

The coated textiles proved suitable for continuous use in wearable applications by retaining their mechanical flexibility and electrical conductivity. In particular, system T3 (whose formulation contained 6 wt% of thickener and 3 wt% of crosslinker) showed the best results in terms of the electrical resistivity value after 120 h of washing cycle. The elastic modulus increased approximately fourfold when ink and coatings were applied to the textile, going from ~20 MPa to ~80–85 MPa in each case.

In order to evaluate the applicative potentiality of the textiles, conductive pathways with a length of 180 mm and a variable width of 5, 3, and 1 mm were tested. The decrease in the conductive section and the subsequent border effects increased the electrical resistivity values.

The piezoresistive behavior demonstrated that the coating decreased the sensitivity by 13% but also enabled the preservation of the long-term usability of the conductive pathways regarding the mechanical stress. The ECG tests showed that the system with a 1 mm width produced unevaluable noisy signals. The electrical resistivity of the coatings increased by one order magnitude without any preference between the three compositions.

Finally, PU-coated e-textiles show promise for a range of applications, especially in healthcare and occupational settings, where dependable, durable, and machine-washable smart textiles are crucial.

## Figures and Tables

**Table 1:** Composition of the coatings.

Coating Name	Crosslinker (wt%)	Thickener (wt%)
T1	2	2.5
T2	2.5	2.5
T3	6	3

**Table 2:** Mechanical properties of pristine PE textile with three layers of ink and coated with the T1, T2, and T3 coatings.

Sample	Tensile Modulus (MPa)	Tensile Strength * (MPa)	Ultimate Strain * (%)
PE	20.2 ± 8.7	6.2 ± 2.0	17.8 ± 2.7
PE/Ink	81.1 ± 0.9	13.8 ± 5.1	16.3 ± 0.6
PE/Ink/T1/5	86.5 ± 8.7	9.8 ± 1.3	13.0 ± 1.7
PE/Ink/T2/5	83.0 ± 13.4	11.0 ± 2.6	14.3 ± 0.9
PE/Ink/T3/5	90.5 ± 6.1	10.1 ± 0.4	12.3 ± 0.3

* Values extracted after the force reached 18 N.

## Data Availability

The original contributions presented in the study are included in the article, further inquiries can be directed to the corresponding author.

## Supplementary Materials

The following supporting information can be downloaded at: https://www.mdpi.com/article/10.3390/polym17070904/s1, Figure S1: Schematic diagram of the screen-printing process for preparing graphene-based wearable e-textiles; Figure S2: Detail of connection between ink conductive pathways and stainless-steel conductive ribbon; Figure S3: TGA with DTGA insert ranging between 200–500 °C (a) and DSC data of heating cycle (b) of pristine TPU (black), T1 (green), T2 (light blue), and T3 (navy blue) films. References [41,44,45] have been cited in the Supplementary Materials.
