# Supplementary material for: On the Enhancement of the Long-Term Washability of e-Textile Realized with Electrically Conductive Graphene-Based Inks
Source: Polymers (Basel). 2025 Mar 27;17(7):904. doi: 10.3390/polym17070904 (PMC11991343; doi:10.3390/polym17070904)
Supplement: Supplementary file 1 [file polymers-17-00904-s001.zip › polymers-3481166-supplementary.pdf]

Supporting Information

On the Enhancement of the Long-Term Washability of e-Textile Realized with Electrically Conductive Graphene-Based Inks

Ilaria Improta [1], Gennaro Rollo [1,*], Giovanna Giuliana Buonocore [1], Simona Del Ferraro [2], Vincenzo Molinaro [2], Gianni D'Addio [3], Anna De Rosa [3,4] and Marino Lavorgna [1]

1 Institute of Polymers, Composites and Biomaterials, National Research Council, 80055 Portici, Italy; ilariaimprota@cnr.it (I.I.); giovannagiuliana.buonocore@cnr.it (G.G.B.); marino.lavorgna@cnr.it (M.L.)
2 INAIL-DiMEILA-Laboratory of Ergonomics and Physiology, 00078 Monte Porzio Catone, Italy; s.delferraro@inail.it (S.D.F.); v.molinaro@inail.it (V.M.)
3 Bioengineering Unit, Institute of Care and Scientific Research Maugeri, 82037 Telese, Italy; gianni.daddio@icsmaugeri.it (G.D.); anna.derosa@icsmaugeri.it or anna.derosa004@studenti.uniparthenope.it (A.D.R.)
4 Engineering Department, University of Naples Parthenope, 80133 Napoli, Italy
* Correspondence: gennaro.rollo@cnr.it

Sample preparation

Screen printing scheme to obtain the electrically conductive pathways:

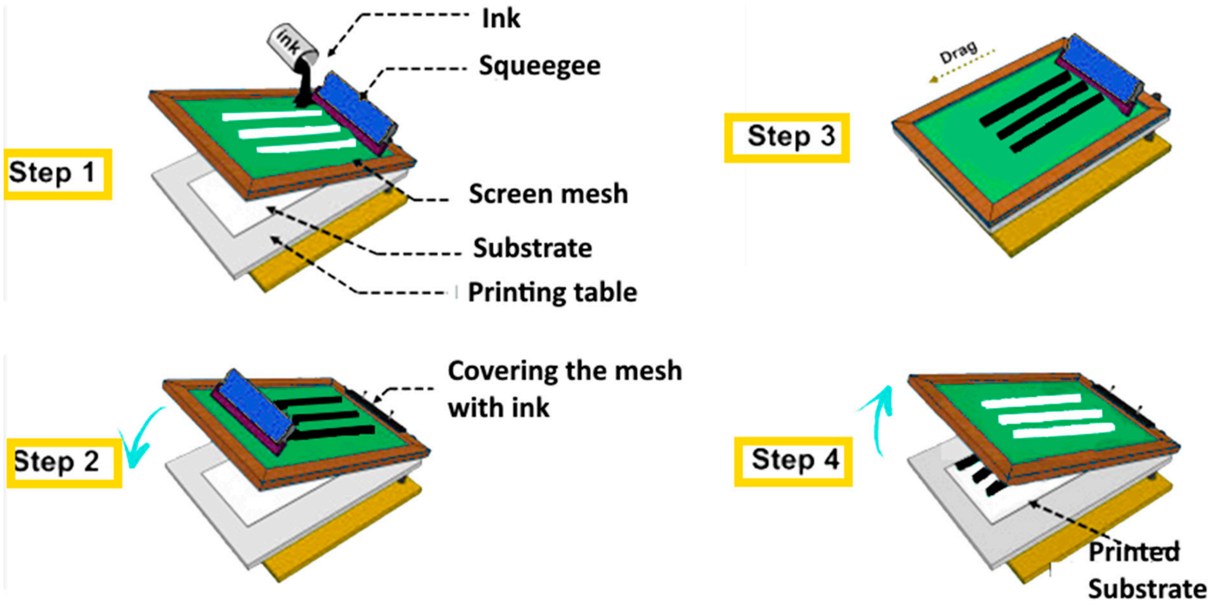

Figure S1. Schematic representation of the screen-printing process.

The conductive tape was attached to the end of the conductive line so that the resistivity value could be read even after applying the insulating coating, as shown in Figure S2.

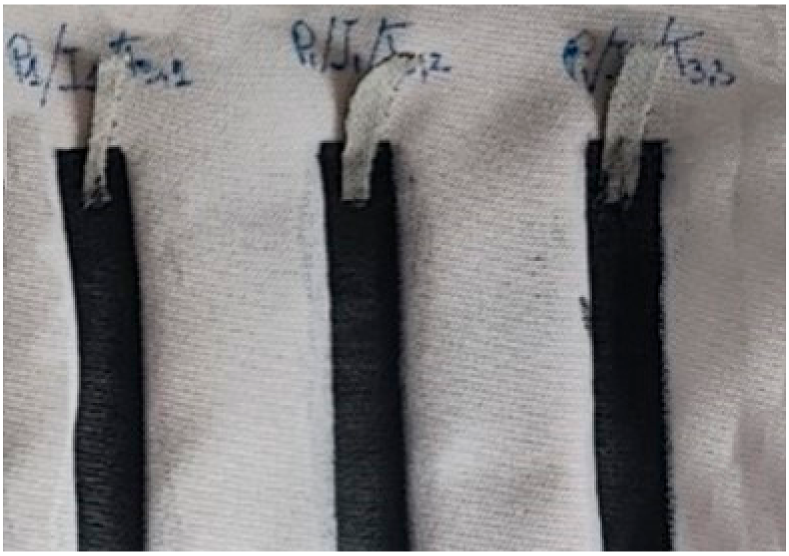

Figure S2. Conductive pathways detail of connection between ink conductive pathways and stainless-steel conductive ribbon.

Thermal Analysis

A thermogravimetric analyser (TGA) (TA Instruments Q500, New Castle, USA) was used to examine the thermal stability of film samples. A nitrogen atmosphere fluxed at 30 mL/min was used to heat each sample, which weighed around 8 mg and had a diameter of 0.5 mm, from room temperature of 25 C to a maximum temperature of 700 C at a rate of 10 C/min. The samples were placed in platinum pans.

The Q2000 Differential Scanning Calorimeter (DSC) (TA Instruments, New Castle, USA) was used to compare the characteristic properties like the temperature of glass transition, crystallization, melting, or crystallinity degree of the composites. The data were analysed using the TA Universal Analysis software in a heat-cool-heat mode. Film samples with a 0.5 mm diameter and a weight of approximately 3 mg were placed in aluminium pans. The analysis methods consisted of three steps. The first step was from -80 C to 180 C, the second step was from 180 C to -80 C, and the third step was from -80 to 200 C; all rises of 10 C/min were carried out.

Figure S3a shows the TGA analysis of pristine TPU and T1, T2, and T3 films. Polyurethane degradation is recognised as a multi-step, complex process, and the least stable parts of the polyurethane chains control the degradation [1]. In pristine TPU, thermal degradation of urethane linkages in the hard TPU segment and polyol groups in the soft TPU segment occurs at 280 C and 440 C, respectively. The initial stage of degradation

is reported to involve the breakdown of urethane linkages into alcohol and isocyanates, potentially leading to the formation of primary and secondary amines [1].

For TPU and its composites, the oxidation of polyethers, which have lower thermal stability, was shown to occur in the first step of the TGA curves (280-440 C). The polyether and polyamide fractions undergo simultaneous thermal and oxidative degradation in the second stage, starting above 440 C [1,2].

By increasing the amount of cross-linking agent in the TPU films, the mobility of the chains is reduced and the curves are shifted to the right, according to the literature [3].

In the DTGA (inset of Figure 4a) a more pronounced shoulder appears in T3 than in T1 and T2 as the percentage of thickener is increased. Thus the primary and secondary amine bonds reappear, bringing the shape of the curve closer to that of the pristine TPU. These results were confirmed by FT-IR analysis.

In Figure S3b the DSC analysis shows a melting peak (Δm) appears at T1 for the pristine TPU. With the addition of the crosslinker and thickener, a melting peak appears with the addition of the two components. This shows the appearance of progressively larger amorphous zones due to the increase in crosslinker, which limits the ability of the chains to organise into ordered crystalline structures, from a value of 1.246 J/g at T1 to 0.960 J/g at T2 [3].

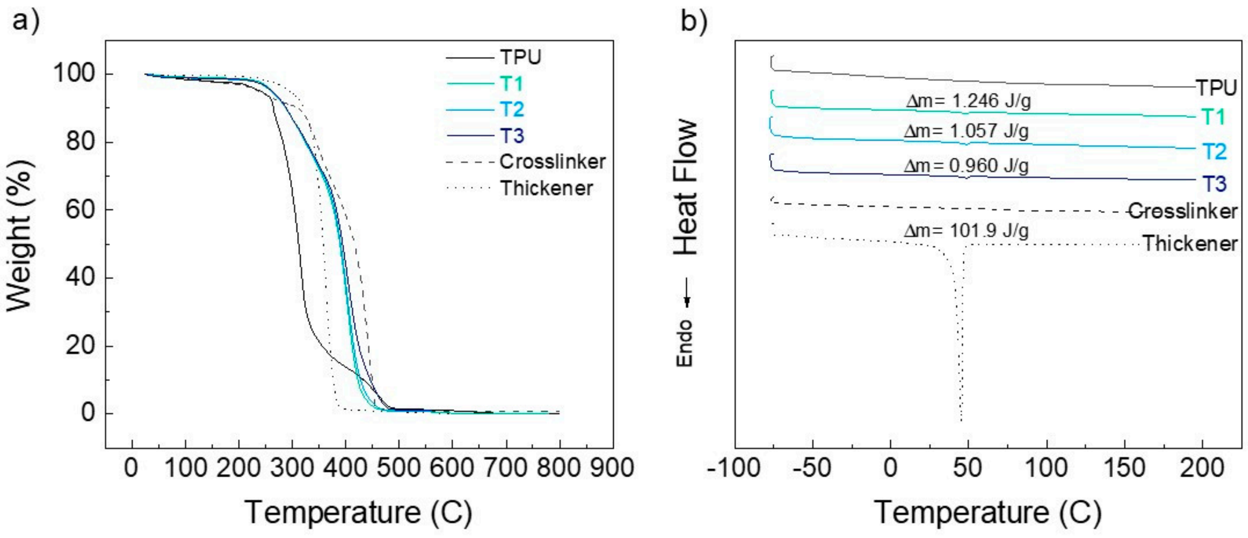

Figure S3. TGA with DTGA insert zooming between 200-500C a) and DSC data heating cycle b) of pristine TPU (black), T1 (green), T2 (light blue) and T3 (navy blue) films.

Reference

1. Bueno-Ferrer, C.; Hablot, E.; del Carmen Garrigós, M.; Bocchini, S.; Averous, L.; Jiménez, A. Relationship between Morphology, Properties and Degradation Parameters of Novative Biobased Thermoplastic Polyurethanes Obtained from Dimer Fatty Acids. *Polym. Degrad. Stab.* **2012**, *97*, 1964–1969, doi:https://doi.org/10.1016/j.polymdegradstab.2012.03.002.
2. Herrera, M.; Matuschek, G.; Kettrup, A. Thermal Degradation of Thermoplastic Polyurethane Elastomers (TPU) Based on MDI. *Polym. Degrad. Stab.* **2002**, *78*, 323–331.
3. Barros Junior, L.P.; de Souza, L.R.; Rahimzadeh, R.; Manas-Zloczower, I. Improving Performance of TPU by Controlled Crosslinking of Soft Segments. *Polym. Eng. Sci.* **2024**, *64*, 3982–3992, doi:10.1002/pen.26826.
